# Supplementary material for: Stable neuronal representations to repeated stimulation underlie cognitive resilience in Alzheimer’s disease pathology
Source: bioRxiv. 2025 Dec 9:2025.12.05.692431. Preprint. [Version 1] doi: 10.64898/2025.12.05.692431 (PMC12713603; doi:10.64898/2025.12.05.692431)
Supplement: 2 [file NIHPP2025.12.05.692431v1-supplement-2.pdf]

bioRxiv preprint doi: https://doi.org/10.64898/2025.12.05.692431; this version posted December 9, 2025. The copyright holder for this preprint (which was not certified by peer review) is the author/funder, who has granted bioRxiv a license to display the preprint in perpetuity. It is made available under aCC-BY 4.0 International license.

Supplementary Figure 1

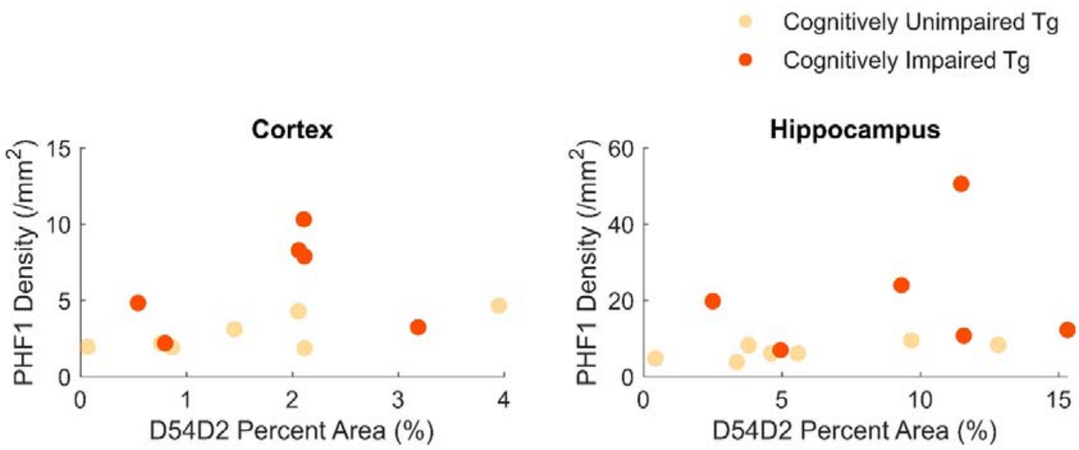

Supplementary Figure 1: Pathological hallmarks are not sufficient to determine cognitive status of Fischer 344 TgAD rats. Scatter plots for Fischer 344 TgAD rats show PHF1-positive inclusion density and D54D2 percent area coverage in the cortex and hippocampus. No clear separation was observed between cognitively unimpaired and impaired groups.

Supplementary Figure 2

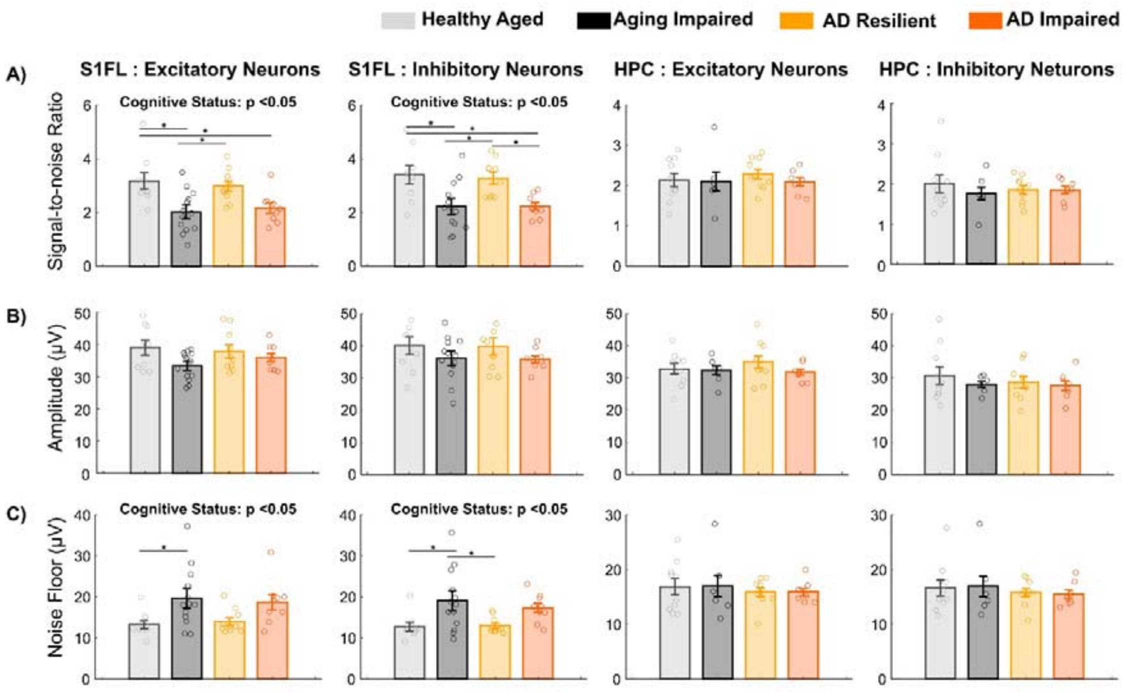

bioRxiv preprint doi: https://doi.org/10.64898/2025.12.05.692431; this version posted December 9, 2025. The copyright holder for this preprint (which was not certified by peer review) is the author/funder, who has granted bioRxiv a license to display the preprint in perpetuity. It is made available under aCC-BY 4.0 International license.

Supplementary Figure 2: **Ultra-High-density single-unit recordings exhibited high SNR in S1FL in cognitively resilient rats.** **A)** S1FL excitatory and inhibitory neurons in 'AD Resilient' (E: 2.99 ± 0.59; I: 3.28 ± 0.68) and 'Healthy Aged' (E: 3.18 ± 0.93; I: 3.41 ± 1.02) rats had significantly higher SNR in than those in 'Aging Impaired' (E: 2.02 ± 0.78; I: 2.22 ± 0.90) and 'AD Impaired' (E: 2.17 ± 0.58; I: 2.23 ± 0.42) rats. **B)** peak-to-peak amplitudes were comparable for excitatory and inhibitory SU signals in S1FL and HPC. **C)** Noise levels in channels with detected SUs in S1FL were significantly lower in 'AD Resilient' (E: 14.01 ± 2.89; I: 13.04 ± 2.01) and 'Healthy Aged' (E: 13.27 ± 2.94; I: 12.77 ± 3.08) rats than in 'Aging Impaired' (E: 19.69 ± 7.33; I: 19.09 ± 7.44) and 'AD Impaired' (E: 18.71 ± 5.48; I: 17.38 ± 3.47) rats.

Supplementary Figure 3

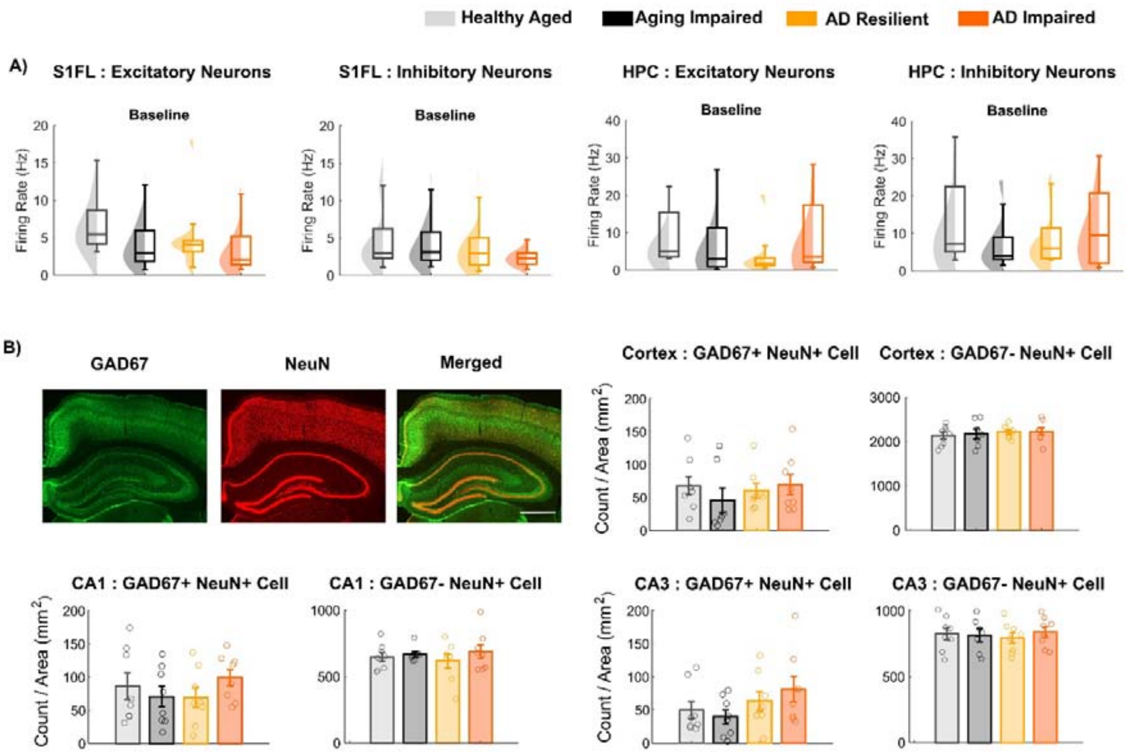

Supplementary Figure 3: Alignment of recording sites and additional characterizations of Single Unit activities. A) The firing rates during pre-stimulation baseline were indistinguishable across the 4 groups for either putative excitatory or inhibitory neurons in S1FL and hippocampus. **B)** Immunostaining-based analysis showed that the densities of GAD67+NeuN+ (inhibitory) and GAD67−NeuN+ (excitatory) neurons in cortex, CA1, and CA3 were comparable across genotypes and cognitive groups. Scale bar = 1 mm.

bioRxiv preprint doi: https://doi.org/10.64898/2025.12.05.692431; this version posted December 9, 2025. The copyright holder for this preprint (which was not certified by peer review) is the author/funder, who has granted bioRxiv a license to display the preprint in perpetuity. It is made available under aCC-BY 4.0 International license.

Supplementary Figure 4

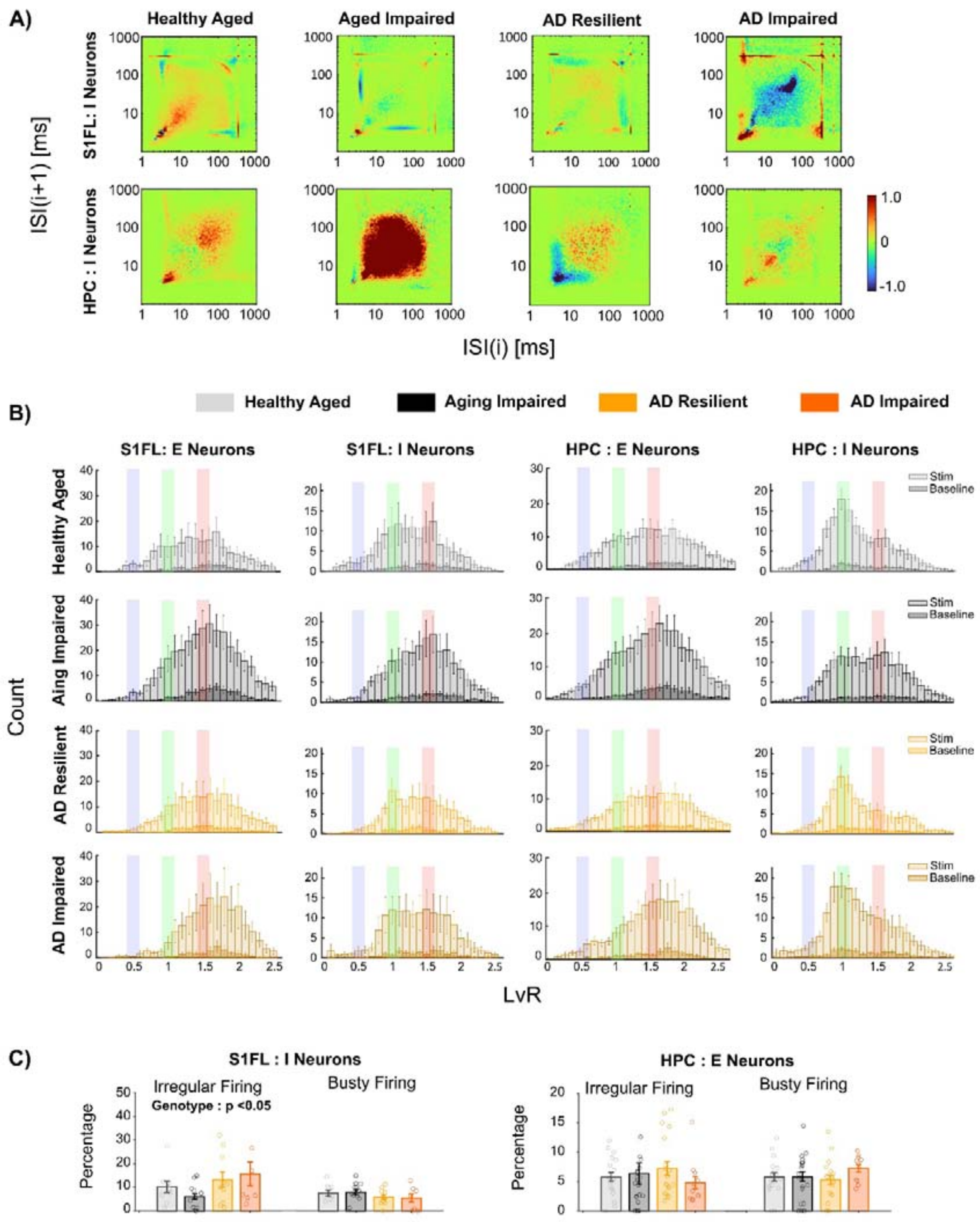

Supplementary Figure 4: Patterns of spike dynamics during network activation. A) In S1FL, 'AD Impaired' rats exhibited a selective reduction in ΔISI spike probability at ~40 ms. In the hippocampus, ΔISI histograms indicated increased burstiness during network activation in 'Aging Impaired' rats. **B)** Histograms of LvR values during forepaw stimulation and pre-stimulation baseline. Colored overlays represent the range of ±0.05 around 0.5 (blue, regular firing), 1.0 (green, random firing), and 1.5 (red, bursty firing). **C)** In S1FL, the percentage of irregular-firing inhibitory neurons (LvR ≈ 1) showed a significant main effect of genotype, with

bioRxiv preprint doi: https://doi.org/10.64898/2025.12.05.692431; this version posted December 9, 2025. The copyright holder for this preprint (which was not certified by peer review) is the author/funder, who has granted bioRxiv a license to display the preprint in perpetuity. It is made available under aCC-BY 4.0 International license.

Tg rats (AD Resilient: 13.21±10.78%, AD Impaired: 15.65±15.02%) exhibiting a significantly higher percentage than nTg rats (Healthy Aged: 10.17±7.47%, Aging Impaired: 6.07±4.69%).

Supplementary Figure 5

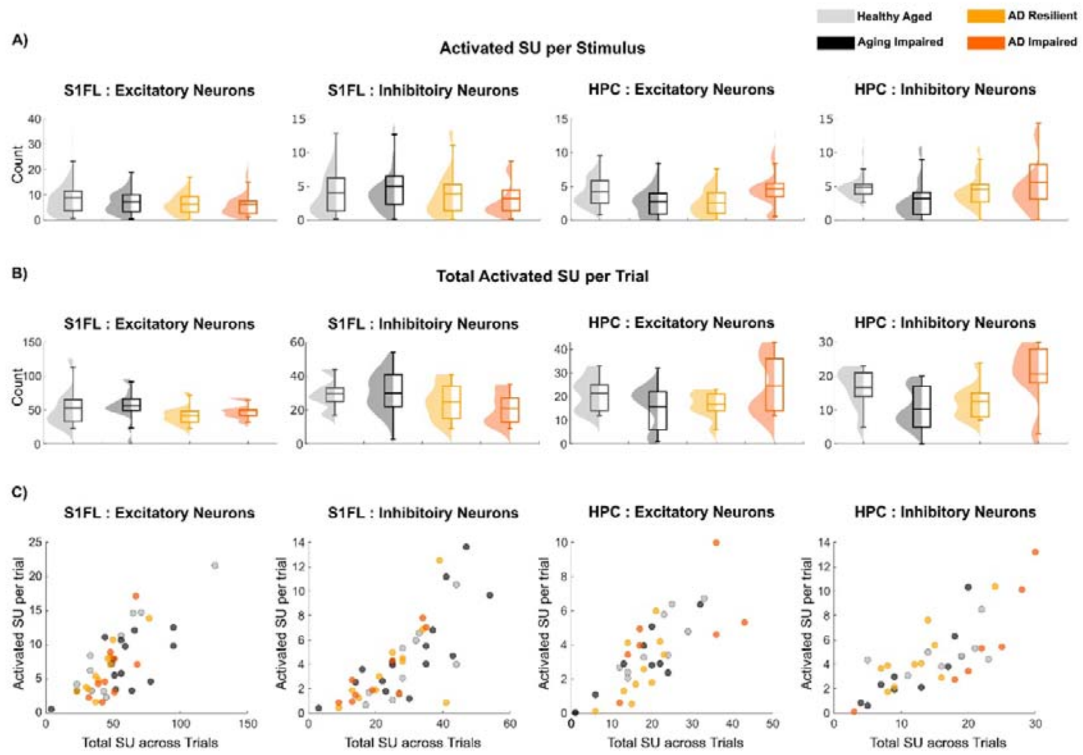

Supplementary Figure 5: Neuronal representation variability. For both putative excitatory and inhibitory neurons in S1FL and hippocampus, **A)** the number of activated single units per stimulus and **B)** The total neurons activated per stimulation trial (with 187 stimuli per trial) were statistically comparable among the four groups. **C)** The mean number of activated SUs per stimulus against the total SU across all trials of each animal.

bioRxiv preprint doi: https://doi.org/10.64898/2025.12.05.692431; this version posted December 9, 2025. The copyright holder for this preprint (which was not certified by peer review) is the author/funder, who has granted bioRxiv a license to display the preprint in perpetuity. It is made available under aCC-BY 4.0 International license.

Supplementary Figure 6:

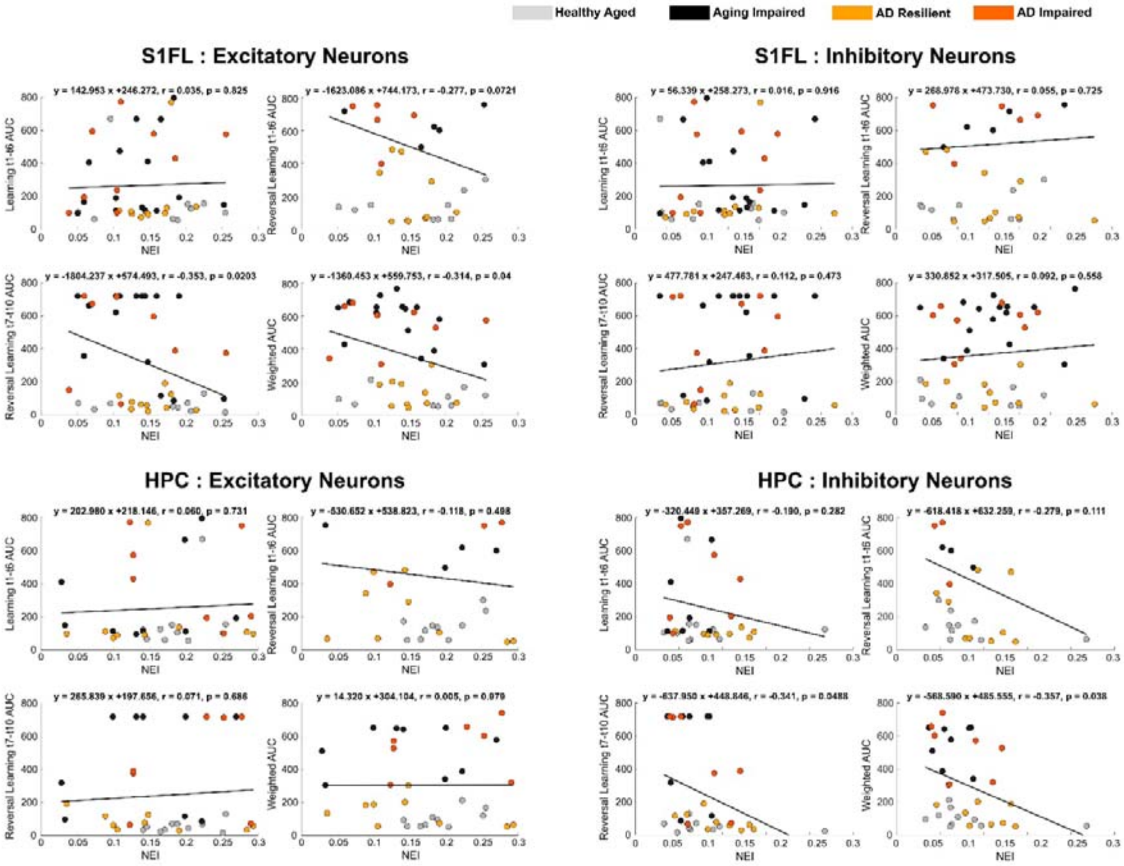

Supplementary Figure 6: Correlation between NEI and Cognitive Performances. Pearson correlation coefficients and regression slopes are shown for the relationships between NEI and the latency AUC metrics (Learning t1–t6, Reversal Learning t1–t6, Reversal Learning t7–t10), as well as a weighted composite score.

bioRxiv preprint doi: https://doi.org/10.64898/2025.12.05.692431; this version posted December 9, 2025. The copyright holder for this preprint (which was not certified by peer review) is the author/funder, who has granted bioRxiv a license to display the preprint in perpetuity. It is made available under aCC-BY 4.0 International license.

Supplementary Figure 7

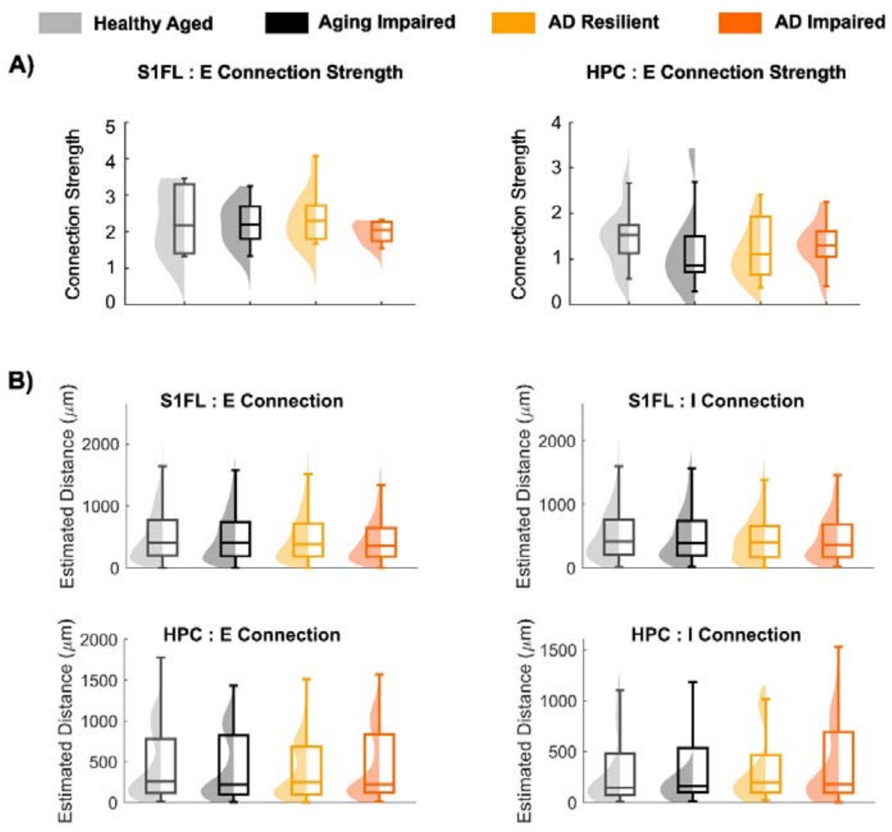

Supplementary Figure 7. Characterization of CCG-based synaptic connections. A) Strength of CCG-based excitatory synaptic connections were comparable across genotypes and cognitive-status groups in both S1FL and hippocampus. **B)** Intersomatic distance between pre- and postsynaptic neurons were estimated from three-dimensional spatial coordinates reconstructed using the high-density recording sites of the Neuropixels probe. No significant differences in the distances between neurons for CCG-based excitatory or inhibitory connection pairs were observed across genotypes or cognitive status in either S1FL or the HPC.

Supplementary Figure 8

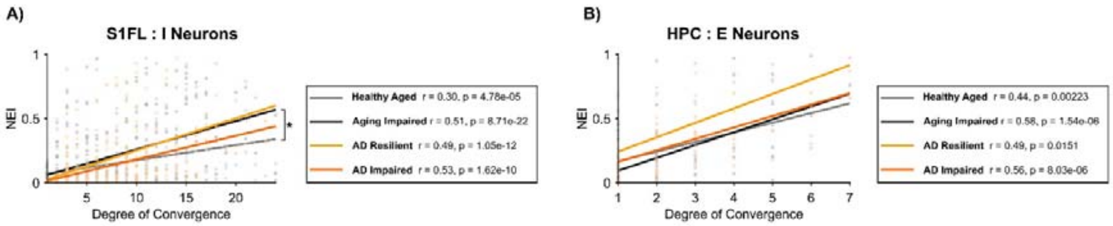

Supplementary Figure 8: Convergence–NEI relationships. A) In S1FL, the linear regression of the degree of convergence onto putative inhibitory neurons vs. their NEI showed a significantly steeper slope in ‘Aging Impaired’ rats vs. in ‘Healthy Aged” rats. **B)** In the hippocampus, the coefficients of Pearson’s correlations

bioRxiv preprint doi: https://doi.org/10.64898/2025.12.05.692431; this version posted December 9, 2025. The copyright holder for this preprint (which was not certified by peer review) is the author/funder, who has granted bioRxiv a license to display the preprint in perpetuity. It is made available under aCC-BY 4.0 International license.

between the degree of excitatory inputs convergent onto HPC excitatory neurons and their NEI were comparable across genotype and cognitive-status groups.
